# *In ovo* MRI of the chicken spleen: feasibility of a new tool for basic research

**DOI:** 10.3389/fvets.2026.1868600

**Published:** 2026-06-29

**Authors:** Yasamin Vali, Eberhard Ludewig, Dieter Liebhart, Sina Bagheri

**Affiliations:** 1 Diagnostic Imaging, Clinical Centre for Small Animal Health and Research, Clinical Department for Small Animals and Horses, University of Veterinary Medicine Vienna, Vienna, Austria; 2 Unit for Poultry Medicine, Clinical Centre for Population Medicine in Fish, Pig, and Poultry, Clinical Department for Farm Animals and Food System Transformation, University of Veterinary Medicine Vienna, Vienna, Austria

**Keywords:** avian spleen, chicken embryo, diagnostic imaging, immune development, immunology research, *in ovo* MRI, spleen volumetry

## Abstract

The avian spleen, as a major secondary lymphoid organ, plays a key role in immune regulation in chickens lacking structured encapsulated lymph nodes. As a secondary lymphoid organ, it provides microenvironment for immune interactions, antigen recognition, and cytokine signaling. Despite its importance, *in vivo* and *in ovo* assessments remain limited, with most data derived from postmortem studies. Magnetic resonance imaging (MRI) offers a non-invasive, high-resolution method for evaluating organ morphology and volume, enabling assessment of morphological changes *intra vitam*. This study evaluated *in ovo* MRI for spleen visualization and volumetry in chicken embryos as a potential tool for immunological and developmental research. Ten SPF chicken eggs were incubated under standardized conditions and imaged on day 18 using a 3 T MRI scanner. Volumetric analysis was conducted on T2W-CISS images using Amira software following manual segmentation of spleen and embryo. Mean spleen volume was 17.72 mm^3^ (SD 1.78; range 14.20–19.37) and mean embryo volume 21,439.27 mm^3^ (SD 944.16; range 19,823.24–23,127.17). Spleen-to-embryo volume ratio was 0.0825 ± 0.0060%. Mean spleen weight was 0.0154 g (SD 0.0046; range 0.008–0.022) and embryo weight 23.5 g (SD 3.37; range 15–25), with weight ratio 0.068 ± 0.025%. MRI volumetry strongly correlated with weight measurements (*r* = 0.96, *p* < 0.01). These findings demonstrate *in ovo* MRI as a feasible, non-invasive tool for splenic morphology and volumetry. It could support studies on infection dynamics, inflammation, and disease processes in avian models, and enable longitudinal evaluation beyond hatching, tracking splenic development and disease progression from embryo to postnatal life in the same individuals over time.

## 1 Introduction

The avian spleen plays a central role in immune defense, particularly in the absence of structured and encapsulated lymph nodes seen in birds like chickens (1). As a secondary lymphoid organ, it provides the crucial environment for interactions between immune cells, antigens, and cytokines, which are necessary for mounting effective immune response. This function becomes especially important during embryonic and early post-hatch stages, when the avian immune system is still maturing and other lymphoid tissues have not yet reached full functional capacity (2). During this period of immune maturation, functional changes are accompanied by measurable alterations in splenic morphology, including variations in size, weight, and structural organization (3). Within this developmental context, the chicken embryo has emerged as a widely used and highly informative model for studying immune ontogeny. Because both innate and adaptive immune mechanisms undergo gradual maturation prior to hatch, the chicken embryo serves as a suitable system for investigating early immune development and immune modulation strategies (4). Consequently, understanding splenic development during these critical early stages is essential for advancing knowledge of avian immunity, disease resistance, and immune regulation.

Despite the central immunological importance of the spleen, assessment of the avian spleen has traditionally relied almost exclusively on postmortem analyses, including gross morphology, histology, and organ weight measurements (3, 5). While informative, these approaches are inherently invasive and therefore preclude longitudinal evaluation within the same individual, thereby restricting the assessment of temporal changes during development and disease (5). As a direct consequent, dynamic processes such as spleen development, immune activation, and responses to environmental or infectious stimuli throughout embryogenesis remain poorly and incompletely characterized (6). Moreover, the lack of non-invasive methodologies has further hindered the ability to directly link structural changes during spleen development with corresponding functional immune outcomes.

To address these limitations, magnetic resonance imaging (MRI) offers a non-invasive, high-resolution imaging modality capable of visualizing soft tissues with excellent contrast. Unlike traditional postmortem approaches, its application in embryonic and developmental research has expanded in recent years, providing a viable alternative for studying organogenesis without compromising embryonic viability. In light of these advances in diagnostic imaging, *in ovo* MRI, in particular, allows repeated visualization of developing organs within the intact egg, thereby enabling longitudinal and quantitative assessment of organ morphology and volume (7). Accordingly, this approach holds significant promise for investigating immune organ development and for establishing imaging-based biomarkers that may link structural changes to immunological and pathological processes. However, it should be acknowledged that the present study is limited by a small sample size and therefore provides only proof-of-concept evidence with limited statistical power, precluding broader biological generalization at this stage.

Micro-MRI approaches have been used to image live avian embryos (e.g., quail) *in ovo*, highlighting the feasibility of high-resolution imaging during early developmental stages and further reinforcing the versatility of MRI in embryonic research (8). Building on this potential, recent advancements in MRI technologies have enabled quantitative analyses of *in ovo* structures such as the yolk sac, amniotic fluid, and the developing embryo, providing longitudinal volumetric data throughout development and underscoring the utility of MRI for precise morphological evaluation in intact eggs (9). MRI has also been applied successfully to serially monitor brain development in chick embryos, demonstrating its capability for longitudinal imaging of soft tissue structures and suggesting potential for broader applications in organogenesis studies (10). The chicken embryo model has long been utilized in developmental studies (4) and sexing (11) and is now increasingly adopted in biomedical research (12), particularly under the ethical framework of the 3Rs (Replacement, Reduction, and Refinement). Encouraged by regulations such as FDA Modernization Act 2.0 (2022, September 29, https://www.congress.gov/bill/117th-congress/senate-bill/5002), the chicken embryo serves as a valuable alternative to traditional animal models. Its application in vascular biology (13), and immunology (14) demonstrates not only its versatility but also its ethical and economic advantages in preclinical research, particularly for studying organ development, vaccine efficacy, and therapeutic response.

Given the early vulnerability of chicks to infectious diseases, timely immune activation is crucial in poultry production. Against this backdrop, early immunization strategies, particularly *in ovo* vaccination, have gained considerable interest for their potential to confer protection before hatch (15, 16). Despite this, the connection between the structural development and functional maturity of the chicken spleen during embryonic and neonatal stages remains poorly understood. Therefore, in this study, we aimed to evaluate the feasibility of assessing the spleen *in ovo* using magnetic resonance imaging (MRI), with the goal of establishing its potential as an early indicator for future immunological experiments.

## 2 Materials and methods

A total of ten fertilized specific pathogen-free (SPF) fertile eggs of layer birds (VALO BioMedia GmbH, Osterholz- Scharmbeck, Germany) were incubated under standard conditions (37 °C, 65% relative humidity). On day 12 of incubation, all eggs were candled to confirm embryo viability. The experimental procedures complied with Directive 2010/63/EU (Directive, 2010) on the protection of animals used for scientific purposes.

**Figure 1:**
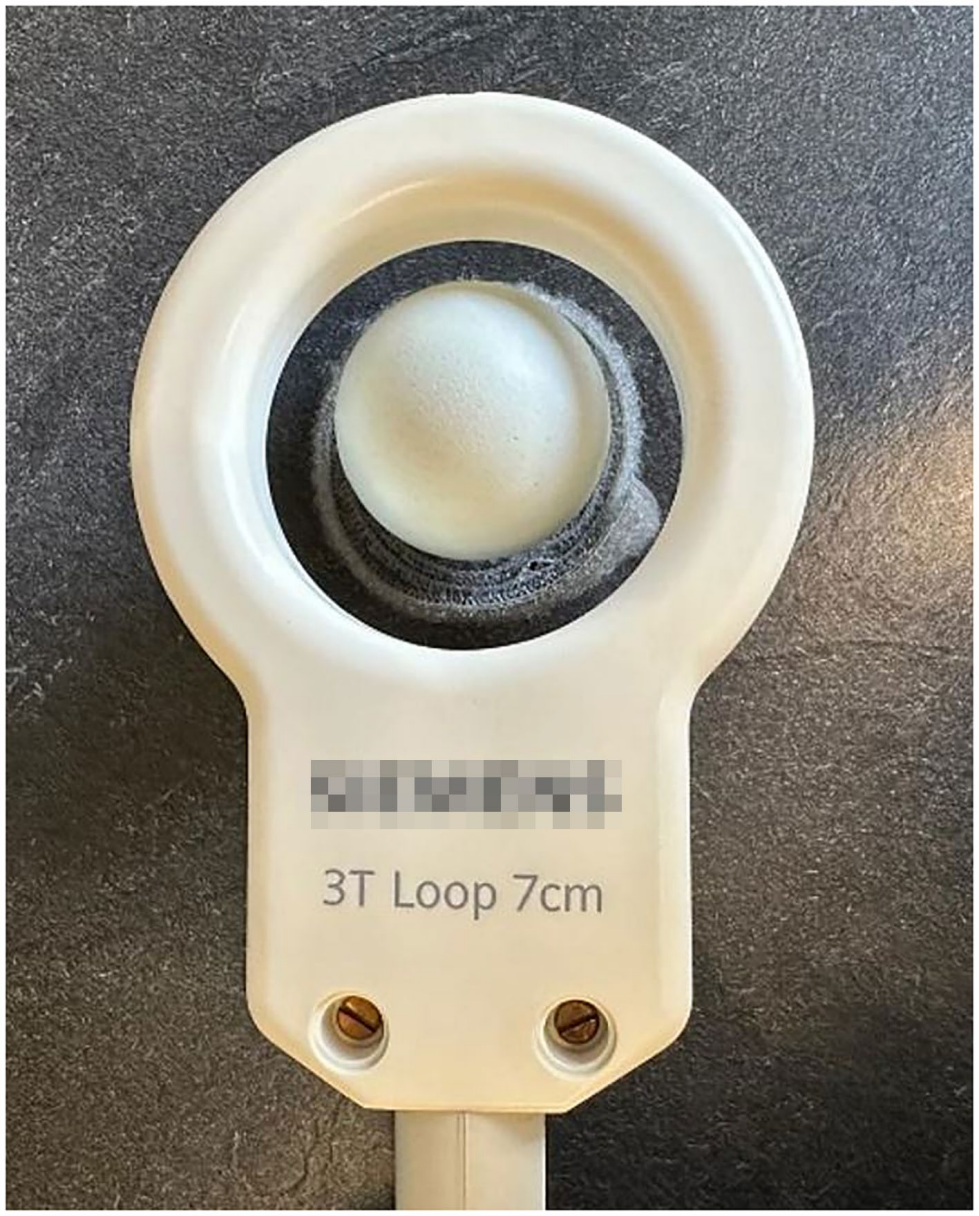
The egg placed on the textile stand in the center of the medium-sized (7 cm) loop coil.

**Figure 2:**
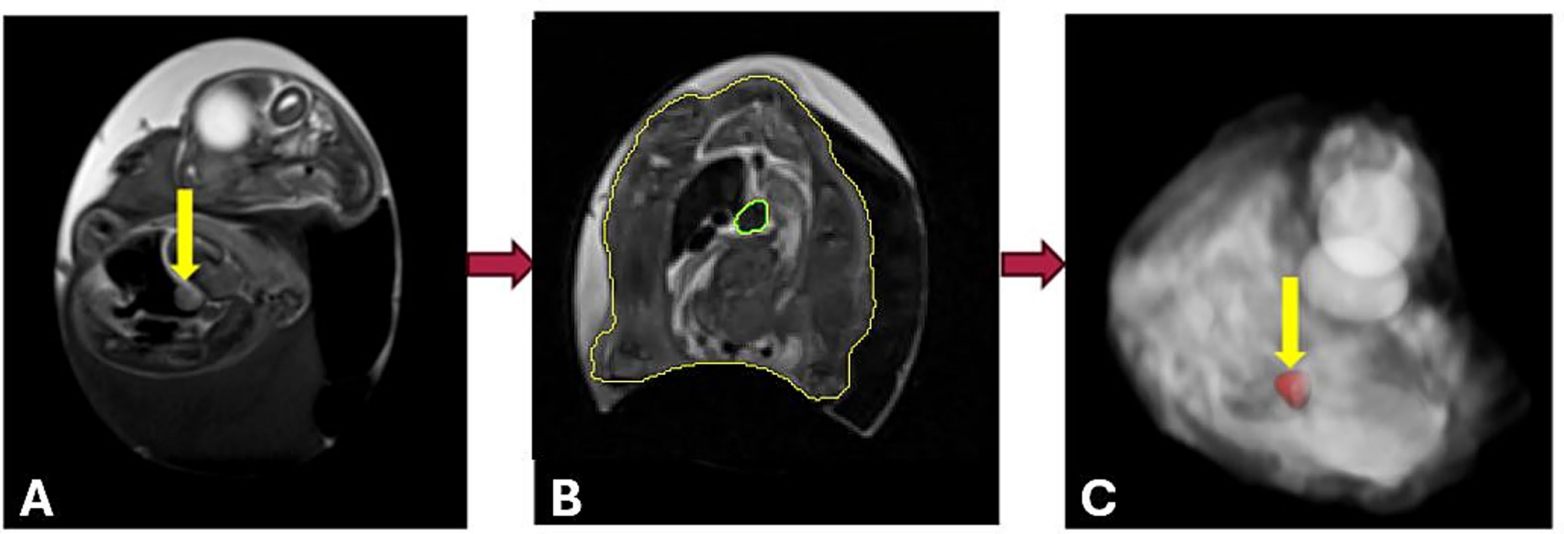
T2W-CISS sequences of *in ovo* chicken embryo. **(A)** Two-dimensional sagittal plane of the embryo, the spleen is marked by arrow. **(B)** Selection of the embryo’s margin and spleen for segmentation and analysis of the volumes. **(C)** Three-dimensional rendering reconstruction of the segmented embryo (transparent) and spleen (red, arrow) for demonstrating the relative ratio.

At day 18 of incubation, the eggs were taken out of the incubator and left for 1 h at room temperature. Then they were cooled down for 15 min prior to MRI in a freezer maintained at −20° C (7). Magnetic resonance imaging (MRI) of the eggs was performed using a Siemens Magnetom Espree 3 Tesla scanner (Siemens, Germany). A medium-sized loop coil with a diameter of 7 cm was employed to ensure optimal image quality. The egg was placed in a paper egg tray and was set at the center of the loop coil (Figure 1). Standardized imaging protocols were applied, including T2-weighted constructive interference in steady-state (CISS) 3D (TR 1200 ms, TE 141 ms, slice thickness 0.5 mm), T1-weighted Volumetric Interpolated Breath-hold Examination (Vibe) transverse (TR 8.36 ms, TE 3.52 ms, slice thickness 0.7 mm), T2-weighted Turbo Spin Echo (TSE) transverse (TR 2690 ms, TE 85 ms, slice thickness 1.5 mm). These protocols ensured consistent data acquisition for all the eggs. The quality of the images was approved by a European Board-certified veterinary radiologist (YV) who also reviewed the sequences for the best visualization of the spleen. T2W-CISS sequences were used for volumetric analysis. DICOM datasets were imported into Amira® (v23.1.1, Thermo Fisher Scientific Inc., Mérignac, France), and the spleen and embryo were manually segmented using the Lasso tool (Figure 2). Noise reduction and smoothing filters (Arithmetic, Gaussian, and surface-smoothing) were applied, and final volumes were calculated automatically with the Material Statistics module.

After the MRI, each egg was weighed prior to opening. Embryos were processed individually, separated from the yolk sac, and embryo weight (excluding the yolk sac) together with spleen weight were recorded. The spleen-to-embryo weight ratio was calculated, and associations were evaluated using Pearson’s rank correlation coefficient (r). Descriptive statistics were performed using GraphPad Prism 10 (GraphPad Software Inc., San Diego, USA). Mean, standard deviation, median and range were calculated where appropriate. A box plot was generated by Microsoft® Excel® for Microsoft 365 MSO (Version 2,508 Build 16.0.19127.20622, Microsoft Corporation, Redmond, WA, USA) to visualize the distribution of the data.

## 3 Results

**Figure 3:**
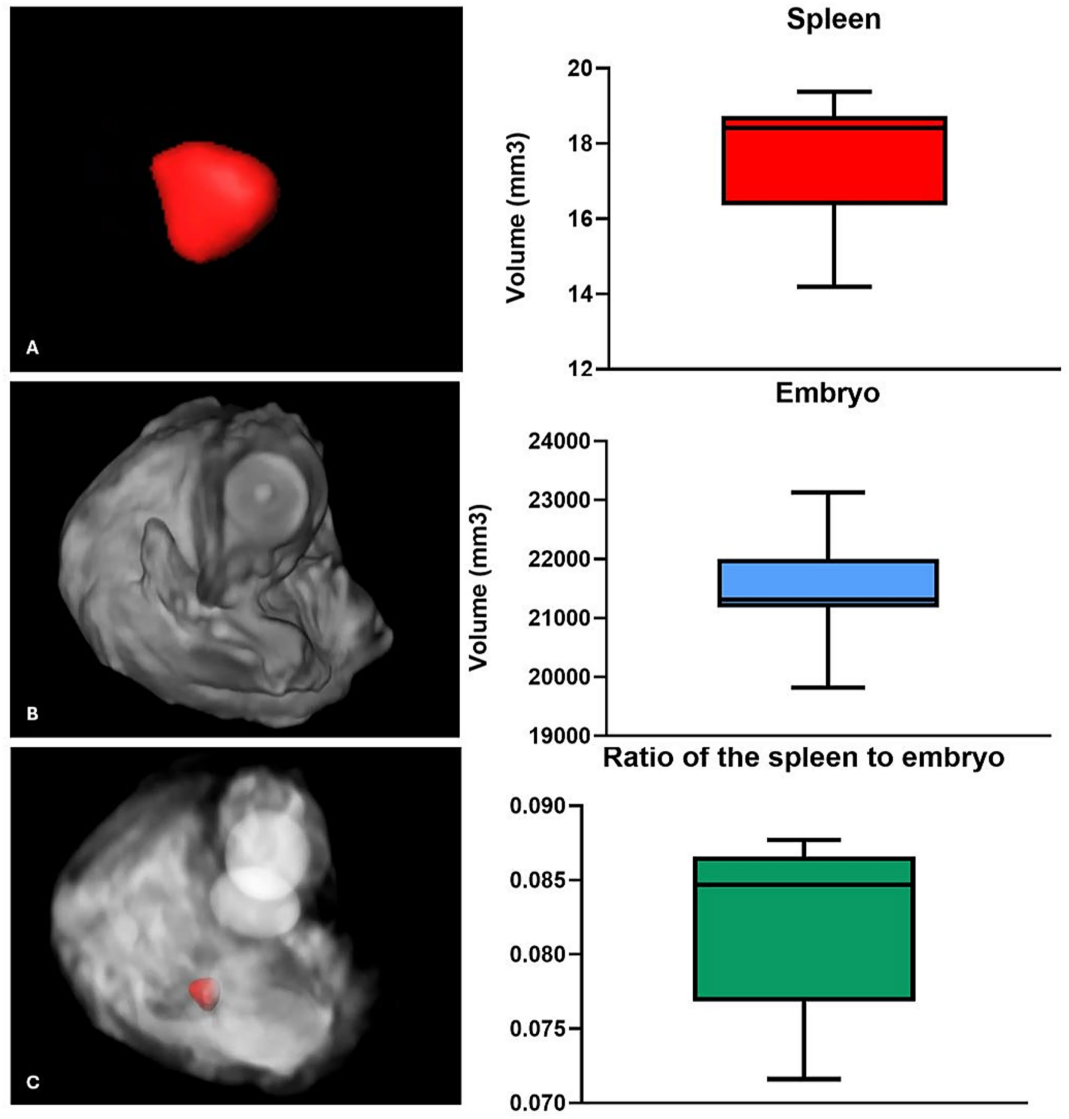
Box plots illustrating *in ovo* volumetric measurements. **(A)** Spleen volume (mm^3^), **(B)** embryo volume (mm^3^), and **(C)** spleen-to-embryo volume ratio. The plots display the median, interquartile range, minimum, and maximum values.

The volumetric analysis demonstrated consistent measurements for both spleens and embryos. The average spleen volume was 17.72 mm^3^, with a median of 18.42 mm^3^, standard deviation of 1.78 mm^3^, and values ranging from 14.20 mm^3^ to 19.37 mm^3^ (Figure 3A). For the embryos, the mean volume was 21,439.27 mm^3^, with a median of 21,310.90 mm^3^, standard deviation of 944.16 mm^3^, and a range from 19,823.24 mm^3^ to 23,127.17 mm^3^ (Figure 3B). Corresponding weight data revealed a mean spleen weight of 0.0154 g (SD = 0.0046 g; range = 0.008–0.022 g) and a mean embryo weight of 23.5 g (SD = 3.37 g; range = 15–25 g), with a mean spleen-to-embryo weight ratio of 0.068 ± 0.025%. A strong positive correlation between MRI-derived volumetric and weight-based measurements (Pearson’s *r* = 0.96, *p* < 0.01) confirmed the reliability of MRI volumetry for estimating true organ size relationships. The spleen-to-embryo volume ratio showed a mean of 0.0825, with a median of 0.0834%, standard deviation of 0.0060%, and a range from 0.0716 to 0.0877 (Figure 3C).

## 4 Discussion

This study provides the evidence that *in ovo* MRI can be applied successfully to monitor spleen volume in chicken embryos. The volumetric data obtained in this study demonstrated measurements of both spleen and embryo volumes, with a relatively narrow distribution of spleen-to-embryo ratios across individuals. These results confirm that MRI is a feasible, non-invasive method for assessing structural changes in the embryonic spleen and lay the groundwork for its application in avian immunology research.

Despite reported cross-sectional imaging splenic volumetry in dogs (17), cats (18), rabbits (19), and humans (20), there is a lack of knowledge regarding volumetric assessment of the chicken spleen *in ovo*. Therefore, direct comparison of the present results with previous studies remains limited. However, the current study is primarily focused on the feasibility and methodological establishment of *in ovo* MRI.

Building on these findings, it is important to consider the functional significance of spleen size. The avian spleen is a key immune organ, and its size has often been considered a potential indicator of immune system activity under various conditions (21, 22). Spleen size has also been proposed as a marker of immune responses in infectious diseases, including avian pathogenic *Escherichia coli* infection, which induces significant splenic immune activation (23, 24). However, the clinical translation of these observations remains limited (21, 25). While spleen evaluation can be performed postmortem through necropsy, diagnostic imaging provides a non-invasive means for antemortem assessment of splenic size, shape, and parenchymal structure. Compared with traditional methods, imaging offers superior diagnostic value by avoiding superimposition of structures and providing enhanced contrast resolution. Previous CT studies in chickens demonstrated low inter- and intra-observer variability in spleen measurements, supporting the reliability of cross-sectional imaging measurements for both routine clinical use and longitudinal follow-up (22).

In mammals, the role of the spleen is well established, and the underlying mechanisms of splenomegaly are well documented (26). However, important interspecies differences must be considered when translating this knowledge to avian species. Unlike mammals, the avian spleen is not involved in erythrocyte storage and contributes only marginally to erythropoiesis (2). Instead, it functions primarily as an immune organ, with spleen size shown to correlate with immunocompetence (6). Because the avian spleen is largely composed of water, descriptors such as spleen size, spleen weight, and spleen volume are often used interchangeably (27, 28). This characteristic makes volumetric assessment particularly informative. In this context, non-invasive imaging techniques such as MRI offer a promising and unique opportunity to monitor spleen development and immune-related changes *in vivo*, effectively bridging the gap between anatomical measurement and functional immunological studies.

Studies of avian immune development have often relied on sampling methods such as flow cytometry (18, 29, 30). Thereby, unchanged immune cell populations assessed by flow cytometry were reported to correlate with uniform spleen sizes or spleen-to-body weight ratios, whereas alterations in these ratios indicated measurable changes in immune cell populations (29, 30). In line with these observations, the relatively consistent spleen-to-embryo ratios observed in the present study reflect the absence of major alterations in splenic immune development in normal embryos. Although flow cytometry remains unique for the detailed characterization of immune cell populations, non-invasive MRI-based spleen volumetry may provide an initial indirect marker of underlying cellular alterations, potentially reflecting changes in immune cell composition or activity. In this context, volumetric findings could help identify embryos for which further targeted flow cytometric analyses are warranted and may support future research exploring correlations between spleen volume, splenic cell quantities, and spleen-to-embryo weight ratios, which require further investigation to clarify their biological relationships. Nevertheless, as a non-invasive approach, MRI-based volumetry may represent an important methodological refinement for experimental studies by enabling longitudinal assessment while potentially reducing the need for invasive sampling, thereby aligning with the 3Rs principles (Replacement, Reduction, and Refinement), which are increasingly emphasized by both European and U.S. regulatory frameworks (31).

The feasibility of non-invasive imaging and volumetry of embryonic organs, as shown in this study, opens up a wide range of new research opportunities in developmental biology, immunology, and infectious disease research. Importantly, this approach is not limited to the spleen; other vital organs, including the liver and brain, can also be monitored longitudinally during embryonic development. Non-invasive imaging enables repeated, real-time assessment of organ growth, morphology, and developmental dynamics without compromising embryo viability. This capability can further be applied for studying the effects of different pathogens, as it may allow detection of pathogen-induced changes in organ size and structure. When used in parallel with complementary techniques such as flow cytometry, histology, or molecular analyses, non-invasive imaging can be integrated with detailed assessments of cellular composition, cell populations, and functional responses. Together, these combined approaches provide a comprehensive framework for investigating host–pathogen interactions, organ maturation, and developmental adaptations during embryogenesis.

In conclusion, this study demonstrates the feasibility of using *in ovo* MRI for non-invasive volumetric assessment of the chicken spleen. This approach parallels established applications of fetal MRI in mammals but is uniquely suited to address avian-specific immunological questions, this approach may help optimize vaccination strategies, early disease detection, and intervention protocols, ultimately supporting improved poultry health and production outcomes.

## Data Availability

The raw data supporting the conclusions of this article will be made available by the authors, without undue reservation.

## References

[1] Jeurissen SH. The role of various compartments in the chicken spleen during an antigen-specific humoral response. Immunology. (1993) 80:29–33. 8244460 PMC1422104

[2] Nagy N Oláh I Vervelde L. "Structure of the avian lymphoid system". In: Kaspers B, Schat KA, Göbel TW, Vervelde L, editors. Avian Immunology. London, UK: Academic Press (2021). p. 11–44.

[3] John JL. The avian spleen: a neglected organ. Q Rev Biol. (1994) 69:327–51. doi: 10.1086/418649, 7972679

[4] Garcia P Wang Y Viallet J Macek Jilkova Z. The chicken embryo model: a novel and relevant model for immune-based studies. Front Immunol. (2021) 12:791081. doi: 10.3389/fimmu.2021.791081, 34868080 PMC8640176

[5] Mast J Goddeeris BM. Development of Immunocompetence of broiler chickens. Vet Immunol Immunopathol. (1999) 70:245–56. doi: 10.1016/s0165-2427(99)00079-3, 10507364

[6] Ceccopieri C Madej JP. Chicken secondary lymphoid tissues-structure and relevance in immunological research. Animals. (2024) 14:2439. doi: 10.3390/ani14162439, 39199973 PMC11350708

[7] Bain MM Fagan AJ Mullin JM McNaught I McLean J Condon B. Noninvasive monitoring of chick development *in ovo* using a 7T MRI system from day 12 of incubation through to hatching. J Magn Reson Imaging. (2007) 26:198–201. doi: 10.1002/jmri.20963, 17659540

[8] Duce S Morrison F Welten M Baggott G Tickle C. Micro-magnetic resonance imaging study of live quail embryos during embryonic development. Magn Reson Imaging. (2010) 29:132–9. doi: 10.1016/j.mri.2010.08.004, 20863641 PMC3006493

[9] Streckenbach F Schön H König J Frank M Langner I Stachs O . Longitudinal volumetric analysis of *in ovo* compartments in chicken eggs using ultra-high-field magnetic resonance imaging. Front Vet Sci. (2024) 11:1450572. doi: 10.3389/fvets.2024.1450572, 39742314 PMC11685116

[10] Zhou Z Chen Z Shan J Ma W Li L Jinyan Z . Monitoring brain development of chick embryos in vivo using 3.0 T MRI: subdivision volume change and preliminary structural quantification using DTI. BMC Dev Biol. (2015) 15:29. doi: 10.1186/s12861-015-0077-6, 26208519 PMC4513430

[11] Jia N Li B Zhu J Wang H Zhao Y Zhao W. A review of key techniques for *in ovo* sexing of chicken eggs. Agriculture. (2023) 13:677. doi: 10.3390/agriculture13030677

[12] Chen L Wang S Feng Y Zhang J Du Y Zhang J . Utilisation of chick embryo chorioallantoic membrane as a model platform for imaging-navigated biomedical research. Cells. (2021) 10:463. doi: 10.3390/cells10020463, 33671534 PMC7926796

[13] Chen L Wang S Feng Y Yu J Coudyzer W Van Ongeval C . Development and characterization of a chick embryo chorioallantoic membrane (CAM) based platform for evaluation of vasoactive medications. Microvasc Res. (2022) 142:104372. doi: 10.1016/j.mvr.2022.104372 35483521

[14] Toledo Fonseca E Menezes De Oliveira Silva F Alcântara D Carvalho Cardoso R Luís Franciolli A Alberto Palmeira Sarmento C . Embryonic development of chicken (*Gallus gallus domesticus*) from 1st to 19th day-ectodermal structures. Microsc Res Tech. (2013) 76:1217–25. doi: 10.1002/jemt.22288, 24019213

[15] Abd El-Ghany WA. *In ovo* vaccination technology: an alternative approach to post-hatch vaccination in modern poultry operations. Microbiol Res. (2025) 16:7. doi: 10.3390/microbiolres16010007, 30654563

[16] Peebles ED. *In ovo* applications in poultry: a review. Poult Sci. (2018) 97:2322–38. doi: 10.3382/ps/pey081, 29617899

[17] Baldo CF Garcia-Pereira FL Nelson NC Hauptman JG Shih AC. Effects of anesthetic drugs on canine splenic volume determined via computed tomography. Am J Vet Res. (2012) 73:1715–9. doi: 10.2460/ajvr.73.11.1715, 23106455

[18] Lee Y Chang D Kim S Oh M Ban J Lee M . Computed tomographic heterogeneous enhancement of spleen in healthy cats: comparing with diffuse infiltrative splenic lesions. Front Vet Sci. (2024) 11:1276984. doi: 10.3389/fvets.2024.1276984, 38812561 PMC11135627

[19] Chernev C Procter T Isaac I Koterwas B Eatwell K Keeble E . Computed tomographic features of the normal spleen in rabbits (*Oryctolagus cuniculus* domesticus). Vet Radiol Ultrasound. (2023) 64:844–50. doi: 10.1111/vru.13282, 37496365

[20] Linguraru MG Sandberg JK Jones EC Summers RM. Assessing splenomegaly: automated volumetric analysis of the spleen. Acad Radiol. (2013) 20:675–84. doi: 10.1016/j.acra.2013.01.011, 23535191 PMC3945039

[21] Smith KG Hunt JL. On the use of spleen mass as a measure of avian immune system strength. Oecologia. (2004) 138:28–31. doi: 10.1007/s00442-003-1409-y, 14576931

[22] Vali Y Gumpenberger M Konicek C Bagheri S. Computed tomography of the spleen in chickens. Front Vet Sci. (2023) 10:1153582. doi: 10.3389/fvets.2023.1153582, 37323833 PMC10267307

[23] Bagheri S Mitra T Paudel S Abdelhamid MK Könnyü S Wijewardana V . Aerosol vaccination of chicken pullets with irradiated avian pathogenic *Escherichia coli* induces a local immunostimulatory effect. Front Immunol. (2023) 14:1185232. doi: 10.3389/fimmu.2023.1185232, 37261344 PMC10227613

[24] Sandford EE Orr M Balfanz E Bowerman N Li X Zhou H . Spleen transcriptome response to infection with avian pathogenic *Escherichia coli* in broiler chickens. BMC Genomics. (2011) 12:469. doi: 10.1186/1471-2164-12-469, 21951686 PMC3190404

[25] Møller AP Erritzøe J. Predation against birds with low Immunocompetence. Oecologia. (2000) 122:500–4. doi: 10.1007/s004420050972, 28308342

[26] Møller AP Erritzøe J Garamszegi LZ. Covariation between brain size and immunity in birds: implications for brain size evolution. J Evol Biol. (2005) 18:223–37. doi: 10.1111/j.1420-9101.2004.00805.x, 15669979

[27] Lahey FH. Diseases of the spleen. Postgrad Med. (1949) 5:380–5. doi: 10.1080/00325481.1949.11693818 18120215

[28] Plavnik I Hurwitz S. Organ weights and body composition in chickens as related to the energy and amino acid requirements: effects of strain, sex, and age. Poult Sci. (1983) 62:152–63. doi: 10.3382/ps.0620152, 6828407

[29] Jansen CA van de Haar PM van Haarlem D van Kooten P de Wit S van Eden W . Identification of new populations of chicken natural killer (NK) cells. Dev Comp Immunol. (2010) 34:759–67. doi: 10.1016/j.dci.2010.02.009, 20188123

[30] Lee Y Lee R Kim J Han YH Hunter C Park J. Comparative analysis of changes in immune cell in the chicken spleen across different ages using flow cytometry. BMC Vet Res. (2024) 20:429. doi: 10.1186/s12917-024-04287-2, 39334332 PMC11438354

[31] Wachsmuth L Mensen A Barca C Wiart M Tristão-Pereira C Busato A . Contribution of preclinical MRI to responsible animal research: living up to the 3R principle. MAGMA. (2021) 34:469–74. doi: 10.1007/s10334-021-00929-w, 34009521 PMC8338837

